# The Biocontrol Root-Oomycete, *Pythium Oligandrum*, Triggers Grapevine Resistance and Shifts in the Transcriptome of the Trunk Pathogenic Fungus, *Phaeomoniella Chlamydospora*

**DOI:** 10.3390/ijms21186876

**Published:** 2020-09-19

**Authors:** Amira Yacoub, Noel Magnin, Jonathan Gerbore, Rana Haidar, Emilie Bruez, Stéphane Compant, Rémy Guyoneaud, Patrice Rey

**Affiliations:** 1 INRAE, UMR 1065 Santé et Agroécologie du Vignoble (SAVE), Institut des Sciences de la vigne et du Vin (ISVV), 33883 Villenave d’Ornon, France; amira.yacoub@inrae.fr (A.Y.); noel.magnin33@gmail.com (N.M.); rana.haidar@inrae.fr (R.H.); emilie.bruez@gmail.com (E.B.); 2 BIOVITIS, 15400 Saint-Étienne-de-Chomeil, France; jonathan.gerbore@sabiovitis.fr; 3 Bioresources Unit, Center for Health & Bioresources, AIT Austrian Institute of Technology GmbH, Konrad Lorenz Strasse 24, 3430 Tulln, Austria; Stephane.Compant@ait.ac.at; 4 Institut des Sciences Analytiques et de Physicochimie pour l‘Environnement et les Matériaux—UMR 5254, Microbial Ecology, Université de Pau et des Pays de l’Adour/E2S UPPA/CNRS, IBEAS Avenue de l’Université, 64013 Pau, France; remy.guyoneaud@univ-pau.fr

**Keywords:** grapevine, biocontrol, trunk disease, *Pythium oligandrum*, transcriptomic analyses

## Abstract

The worldwide increase in grapevine trunk diseases, mainly esca, represents a major threat for vineyard sustainability. Biocontrol of a pioneer fungus of esca, *Phaeomoniella chlamydospora*, was investigated here by deciphering the tripartite interaction between this trunk-esca pathogen, grapevine and the biocontrol-oomycete, *Pythium oligandrum*. When *P. oligandrum* colonizes grapevine roots, it was observed that the wood necroses caused by *P. chlamydospora* were significantly reduced. Transcriptomic analyses of plant and fungus responses were performed to determine the molecular events occurring, with the aim to relate *P.* *chlamydospora* degradation of wood to gene expression modulation. Following *P. oligandrum*-root colonization, major transcriptomic changes occurred both, in the grapevine-defense system and in the *P. chlamydospore*-virulence factors. Grapevine-defense was enhanced in response to *P. chlamydospora* attacks, with *P. oligandrum* acting as a plant-systemic resistance inducer, promoting jasmonic/ethylene signaling pathways and grapevine priming. *P. chlamydospora* pathogenicity genes, such as those related to secondary metabolite biosynthesis, carbohydrate-active enzymes and transcription regulators, were also affected in their expression. Shifts in grapevine responses and key-fungal functions were associated with the reduction of *P. chlamydospora* wood necroses. This study provides evidence of wood fungal pathogen transcriptional changes induced by a root biocontrol agent, *P. oligandrum*, in which there is no contact between the two microorganisms.

## 1. Introduction

Applying biocontrol agents (BCAs) to plants can be useful to control diseases, particularly when chemical pesticides have been banned or do not exist [1,2]. This is the case for grapevine trunk diseases (GTDs), which have increased worldwide over the last two decades following the ban, in Europe in the early 2000s, of sodium arsenite, due to its toxic effect on the environment and humans [1,3]. GTDs, especially esca, cause considerable economic losses to the viticulture sector [4], with decreases in plant productivity and longevity, and wine quality alteration [5,6]. The worldwide annual incidence and cost for the replacement of dead plants due to GTDs is very high. In Italy, for instance, depending on the cultivars, the incidence of GTDs ranged from 8% to 19% and around 10% in Spain; in France, approximately 12% of French vineyards are unproductive during the period of 2012-2017 [7], with losses, in 2014, estimated at around € 1 billion.

In order to control esca, various beneficial microorganisms, used as BCAs, have been tested to control the fungal pathogens associated with this disease [1,3,8]. Two biocontrol products called Esquive WP^®^ and Vintec^®^ based on two different strains of *Trichoderma atroviride*, I-1237 and SC1, respectively, have been registered to control esca on the adult grapevine [9,10]. However, their protection levels usually vary from year to year. Consequently, the few BCAs available against GTDs, particularly esca, need their levels of protection to be optimized. Improving our understanding of the molecular mechanisms involved in a defined grapevine, esca-pathogen and biocontrol agent interaction would facilitate the development of this control method.

Among the potential microbial BCAs, we have selected here the oomycete, *Pythium oligandrum*, because it is naturally present in the rhizosphere of many plants, including vineyards [11], and has shown potential in controlling many plant pathogens [12,13]. As regards the plant protection provided by *P. oligandrum*, it results from: (1) the direct effect of the oomycete on the pathogens by destroying them via mycoparasitism, antibiosis and/or competition for nutrient elements and (2) the indirect effect *P. oligandrum* has on plants by inducing host resistance. Scientists generally considered plant-induced resistance as the main mode of action of *P. oligandrum* [13]. Three elicitin-like proteins produced by *P. oligandrum*, such as oligandrin, POD1 and POD2, are also able to trigger this host-resistance when they are applied on plants.

Regarding grapevines, previous studies showed that treating *Vitis vinifera* L. roots with *P. oligandrum* was associated with a reduction in *Botrytis cinerea* infection at the leaf level [14]. It also reduced the wood attacks of two fungal pathogens involved in GTDs [15], *Phaeomoniella chlamydospora* and *Neofusicoccum parvum* [16,17]. Hence, the use of a BCA to control esca could well prove an interesting approach for growers in developing sustainable viticulture.

Various molecular aspects of a grapevine, esca-pathogen and biocontrol agent interaction will be investigated here, since little is known about the molecular events associated with GTD pathogens. The complete genome sequencing of several GTD pathogens [18,19,20] has also paved the way for obtaining molecular insights about the genes involved in the pathological process. Moreover, this enables improved understanding of the plant resistance induced by the BCA.

In the present study, our objective is to extensively decipher the tripartite interaction between the grapevine and a trunk fungal pathogen involved in esca, *P. chlamydospora* and *P. oligandrum*, as a biocontrol agent colonizing the root system, using transcriptomic analyses. First, grapevine microarrays have been used to study the grapevine transcriptomic responses associated with the trunk-infection by the pathogen, *P. chlamydospora*, and root-inoculation with *P. oligandrum*, the main aim being to identify grapevine defense genes specific to this tripartite interaction. Then, the indirect effect of *P. oligandrum*-root colonization on the transcriptome of the wood pathogen, *P. chlamydospora* was studied by RNAseq. This complementary approach using two methodologies provides useful information both on the transcriptomic changes induced in the grapevine and pathogen. To the best of our knowledge, this is the first experiment that studies trunk pathogen transcriptome shifts indirectly induced by a BCA, inoculated at the root level.

## 2. Results

### 2.1. Evaluation of Grapevine Transcriptomic Responses

Differentially expressed (DE) genes in the plant wood tissues were investigated using a time- frame approach with *V. vinifera* microarrays (0- vs. 14-dpi corresponding to 2 h and 14 days after pathogen infection).

#### 2.1.1. Grapevine Trunk Transcriptional Response to *P. oligandrum* Root Colonization

**Figure 1:**
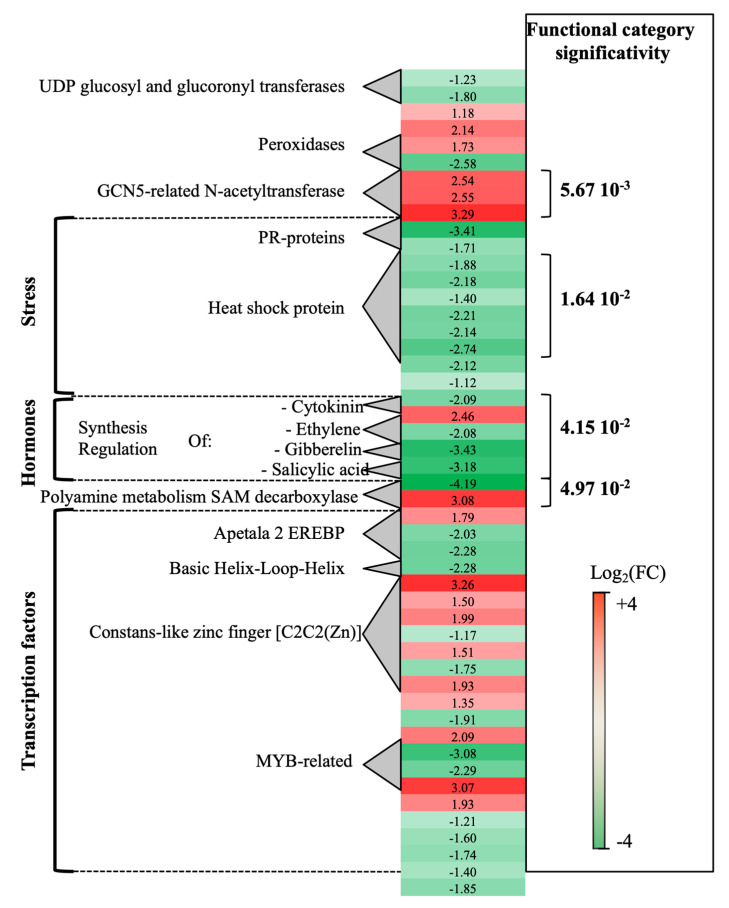
Global aspects of the grapevine transcriptome evolution from 0- to 14-dpi according to the treatments. Major functional categories obtained after Limma analysis and Mapman classification for root-inoculated grapevine with *Pythium oligandrum*, in the absence of pathogen infection. The red color means overexpression at 14-dpi and the green color means overexpression at 0-dpi.

Transcriptomic analysis of *P. oligandrum*-treated plants at 0- vs. 14-dpi revealed that a total of 189 genes were differentially expressed. Seventy percent of these genes (green genes) were overexpressed at 0-dpi. A few functional categories showed statistical significance (Figure 1). Stress-associated genes showed overexpression at 0-dpi (PR-proteins and heat shock protein). On the contrary, polyamine metabolism (two S-adenosylmethionine decarboxylase genes, SAM) and three GCN5-related-N-acetyltransferase associated genes showed overexpression at 14-dpi. As regards the hormone metabolism category, whereas DE genes related to ethylene (ET), gibberellin and salicylic acid were induced at 0-dpi, those concerning cytokinin were overexpressed at 14-dpi.

Other important functional categories, albeit of lesser statistical significance, concerned transcriptional regulation factors: apetala2 ethylene-responsive element binding protein transcription factor family (AP2-EREBP), which was overexpressed, at 0-dpi (3 genes), and basic helix-loop-helix type transcription factor (bHLH), overexpressed at 14-dpi (1 gene).

#### 2.1.2. Transcriptomic Changes in Grapevine Wood Tissues Following *P. chlamydospora* or *P. oligandrum* + *P. chlamydospora* inoculations

**Figure 2:**
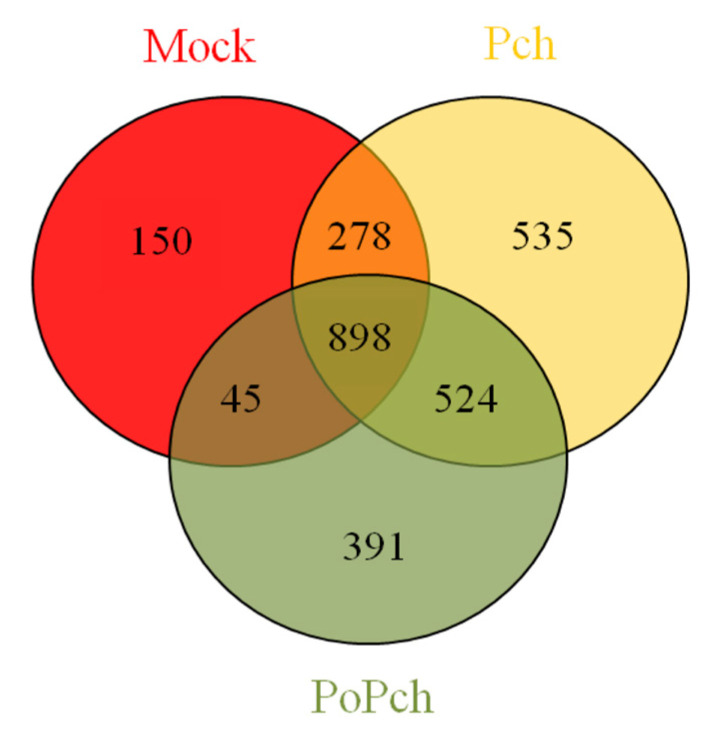
Venn diagram showing gene numbers in grapevine wood tissues common or specific to the three conditions considered ((i) mock inoculation, (ii) *Phaeomoniella chlamydospora-*wood infection, (iii) *Phaeomoniella chlamydospora-*wood infection and *Pythium oligandrum* Po2-root inoculation). Mock: mock inoculation; Po: *P. oligandrum*; Pch: *P. chlamydospora*; PoPch: *P. oligandrum* and *P. chlamydospora*.

In order to evaluate grapevine transcriptomic changes, plant responses to the different treatments (mock inoculation, *P. chlamydospora* and *P. oligandrum* + *P. chlamydospora*) were compared. DE genes between 0- and 14-dpi were obtained using Limma analysis (*p* < 0.01), which gave the following results: mock treatment (1371 genes), pathogen treatment (2235 genes) and pathogen treatment in the presence of the root BCA (1858 genes). The number of specific and common DE genes detected is shown in the Venn diagram (Figure 2). Specific genes for each treatment were thus obtained, and their biological significance compared (Mapman functional category enrichment).

The combined effect of all treatments revealed numerous DE genes (898), and analysis by functional categories using Mapman software indicated that the amplitude of expression appeared to play a major role. Although many interesting features can be obtained from these microarray result analyses, it is important to focus on the optimal points in high-throughput data regarding the biological system under investigation. In our case, these concerned (1) *V. vinifera* defense induction by *P. chlamydospora* and (2) *V. vinifera* induced resistance by *P. oligandrum*. The expected differences among treatments at the induction level for the most important DE genes, and statistically significant functional categories related to induced systemic resistance, ISR, (hormones, secondary metabolites, transcription factors and stress related genes), were effectively found. These results were observed when the genes were considered as common to all treatments, rather than when they were considered as specific to each treatment. Our analysis compared the expression of genes at 0-dpi against those at 14-dpi. Overexpression, for the most important functional categories involved in the establishment of ISR, took place at 0-dpi.

**Figure 3:**
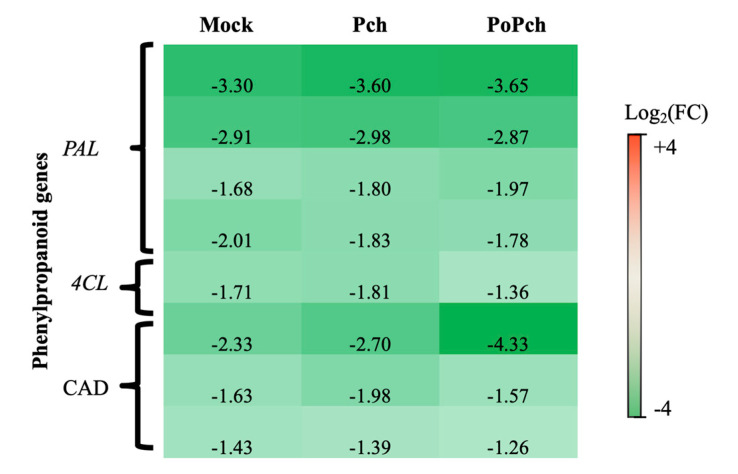
Characteristics of the grapevine transcriptome evolution for genes involved in the phenylpropanoid biosynthesis pathway between 0- and 14-dpi. Expression level values of phenylpropanoid genes (*PAL*: phenyl-alanine-ammonia lyase, *4CL*: 4-coumarate-CoA ligase and *CAD*: cinnamyl alcohol dehydrogenase) are given as the fold change on a log_2_ basis. The red color means overexpression at 14-dpi and the green color means overexpression at 0-dpi. Mock: mock inoculation; Po: *Pythium oligandrum*; Pch: *Phaeomoniella chlamydospora*; PoPch: *P. oligandrum* and *P. chlamydospora*.

In all conditions where wounding occurred (mock inoculation and *P. chlamydospora* infection), the functional category presenting the strongest statistical significance was “secondary metabolism”. More detailed analyses revealed that several genes involved in phenylpropanoid biosynthesis (Figure 3) presented overexpression at 0-dpi (green genes) in the different treatments. These genes included four phenyl-alanine-ammonia lyase genes (*PAL*), one gene for 4-coumarate-CoA ligase (*4CL*) and three cinnamyl alcohol dehydrogenase (*CAD*) genes. One of the *CAD* genes was highly overexpressed at 0-dpi in plants treated with both microorganisms.

**Figure 4:**
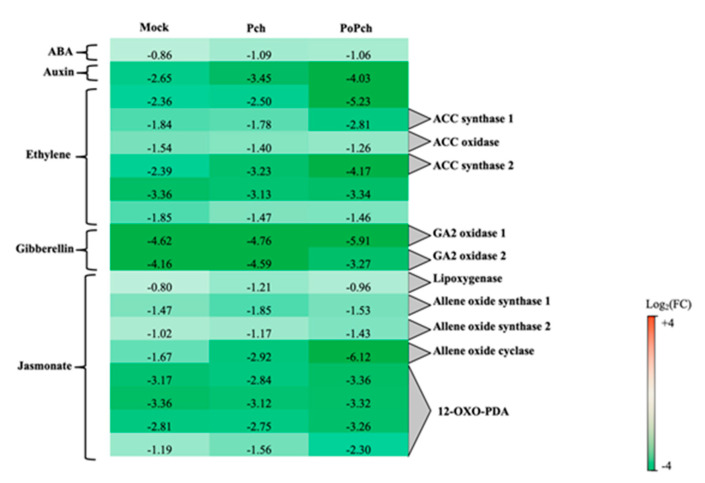
Characteristics of the grapevine transcriptome evolution for genes involved in hormone biosynthesis pathways between 0- and 14-dpi. Expression level values of genes encoding proteins involved in the synthesis or regulation of hormones (*ABA*: abscisic acid, *ACC*: 1-aminocyclopropane 1-carboxylate and *GA*: gibberellin) are given as the fold change on a log_2_ basis. The red color means overexpression at 14-dpi and the green color means overexpression at 0-dpi. Mock: mock inoculation; Po: *Pythium oligandrum*; Pch: *Phaeomoniella chlamydospora*; PoPch: *P. oligandrum* and *P. chlamydospora*.

The “hormone” functional category was statistically significant for all treatments considered, with the jasmonate functional category having the largest number of DE genes (51) in the case of plants inoculated by both *P. oligandrum* and *P. chlamydospora.* Selected genes common to all treatments are presented in Figure 4. Two genes were particularly overexpressed at 0-dpi, but only in the joint presence of *P. oligandrum* and *P. chlamydospora*. One of the genes was involved in ET hormone regulation (log_2_ (FC) = −5.23), and the other one, annotated as allene oxide cyclase (log_2_(FC) = −6.12), was involved in the jasmonic acid (JA) biosynthesis pathway. Seven other genes of that pathway were induced at 0-dpi at various levels. One was lipoxygenase, two allene oxide synthase, and four related to 12 oxo-phytodienoic acid reductase, thereby confirming the strong involvement of the jasmonate pathway in the *P. oligandrum* induction of plant defenses.

**Figure 5:**
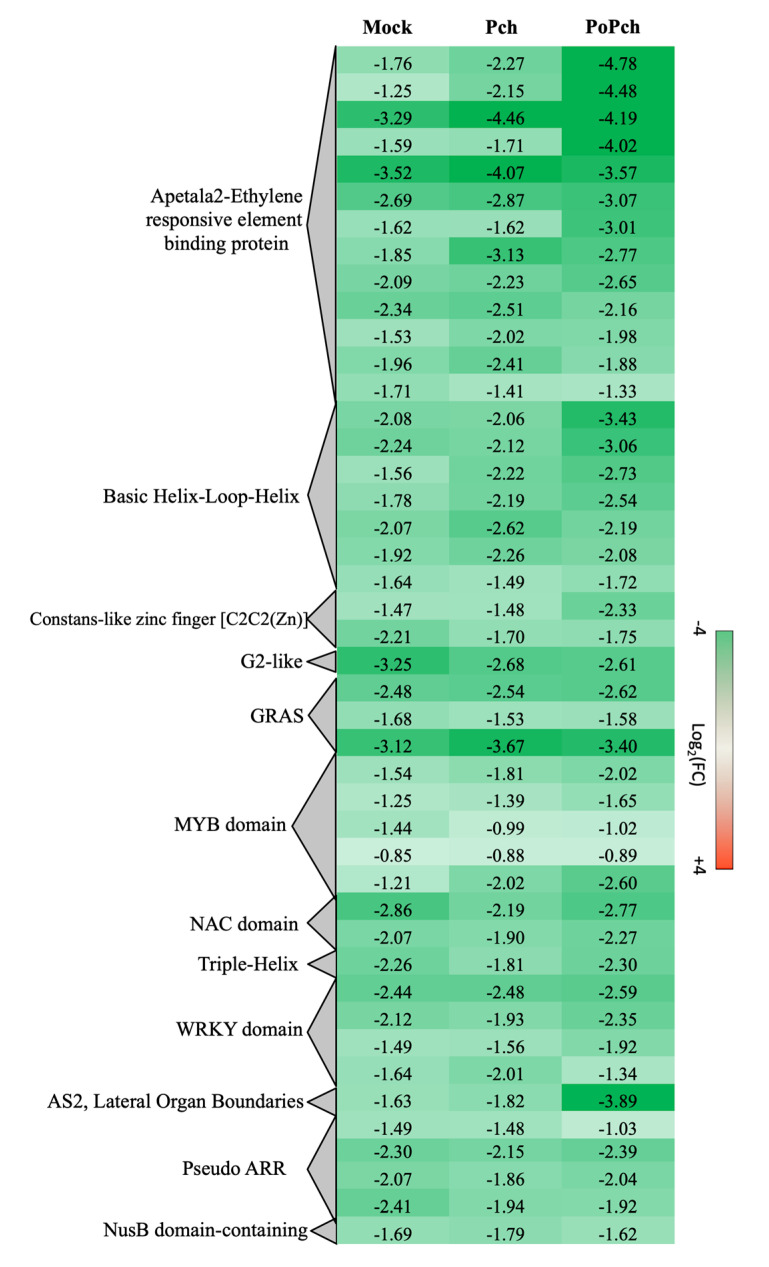
Global aspects of the grapevine transcriptome evolution for the transcription factor functional category between 0- and 14-dpi. Expression level values are given as a fold change on a log_2_ basis. The red color means overexpression at 14-dpi and the green color means overexpression at 0-dpi. Mock: mock inoculation; Po: *Pythium oligandrum*; Pch: *Phaeomoniella chlamydospora*; PoPch: *P. oligandrum* and *P. chlamydospora.*

Two other functional categories, stress-related genes and transcription factors, presented the induction of numerous genes in all treatments at 0-dpi. In the case of stress-related genes induction of abiotic- (heat shock proteins, wounding) and biotic-related genes (pathogenesis-related proteins and receptors), slight differences were detected according to the treatment. However, for transcription factors (Figure 5), *P. oligandrum* + *P. chlamydospora* treated plants showed globally a higher gene induction at 0-dpi than those inoculated with *P. chlamydospora*. Numerous functional categories were presented such as the Apetala2-Ethylene responsive element-binding protein (AP2-EREBP), Basic Helix-Loop-Helix (bHLH), MYB domain and WRKY domain. Moreover, AP2-EREBP was the most represented family, with several genes showing a stronger induction in the presence of *P. oligandrum* and *P. chlamydospora*.

### 2.2. Evaluation of P. chlamydospora Transcriptomic Responses 

RNAseq experiments were designed to compare samples treated with the *P. chlamydospora* pathogen in the presence or not of the BCA, *P. oligandrum*, 14 days after pathogen infection.

**Figure 6:**
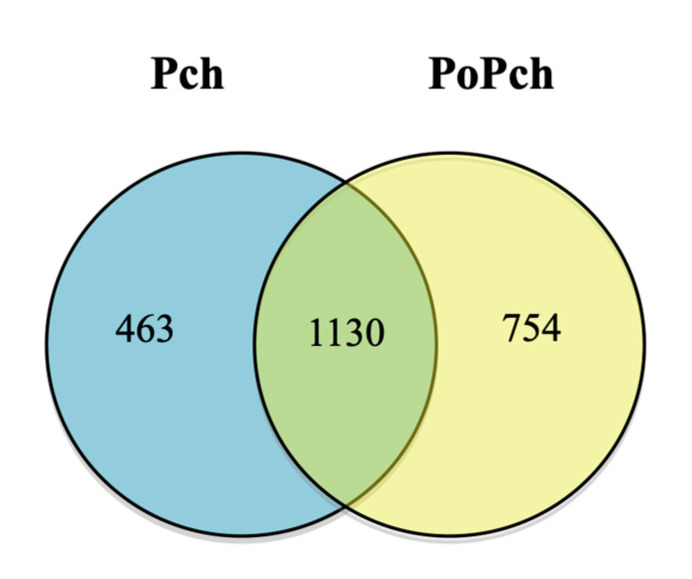
*Phaeomoniella chlamydospora* transcripts detected by RNAseq analysis in grapevine wood tissues according to the treatment (Pch: *P. chlamydospora*; PoPch: *Pythium oligandrum* and *P. chlamydospora*).

The *P. chlamydospora* genome used in our analysis is a draft comprising of 7,279 genes annotated on a BlastP analysis [18]. A relatively high number of transcripts (2347—32% of the total predicted genes) were detected in our RNAseq data: 1,130 different transcripts in both treatments, 754 transcripts specific to the pathogen + BCA treatment and 463 transcripts to the pathogen treatment only (Figure 6). Of the sequences detected in both treatments, 573 were overexpressed and 557 underexpressed in the *P. chlamydospora* + *P. oligandrum* treatment. A more detailed annotation was undertaken in order to obtain further information about the biological signification of our RNAseq data.

**Figure 7:**
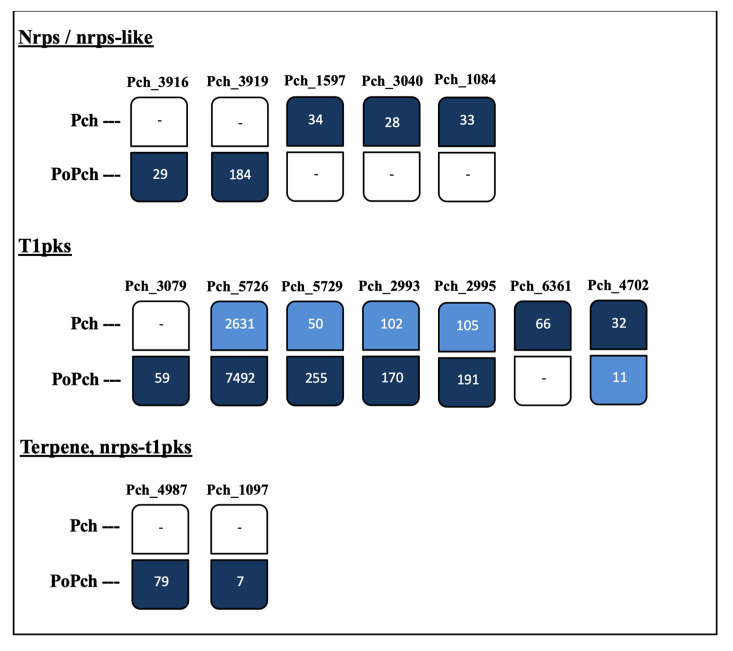
Expression level of *Phaeomoniella chlamydospora* genes predicted as secondary metabolites producers obtained in the RNAseq experiment. Expression level values are given as FPKM (fragments per kilobase per million mapped fragments). nrps = non-ribosomal peptide synthetase; t1pks = type 1 polyketide synthase. Pch: *P. chlamydospora*; PoPch: *Pythium oligandrum* and *P. chlamydospora*. Light blue means that the number of transcripts is lower than in the other treatment. Dark blue means the number of transcripts is higher than in the other treatment. Blank means no transcripts detected in the samples.

The peptide fasta file [18] was used to obtain precise annotations concerning the presence of carbohydrate-active enzymes (CAZymes), and also of peptides involved in secondary metabolite production. The genes potentially involved in secondary metabolite production and detected in our experiment are given in Figure 7. The “Antismash” analysis found gene clusters involved in terpene synthesis: five genes concerned non-ribosomal peptide synthesis (Nrps), and seven others were attributed to type I polyketide synthases (T1pks). In our RNAseq data, five sequences belonging to T1pks clusters were expressed in both treatments (pathogen/BCA and pathogen). Four of these were expressed at a higher level in the pathogen + BCA treatment. Genes expressed only in the pathogen + BCA treatment belonged to the Nrps, T1pks, terpene and Nrps-t1pks hybrid clusters, whereas genes expressed only in the pathogen treatment were restricted to the Nrps and T1pks.

Genes annotated as CAZymes, genes implicated in the degradation of plant polysaccharides and divided into six groups [21], were detected in our samples (Table 1). Most sequences predicted in the *P. chlamydospora* genome belonged to the glycoside hydrolase (GH) families (345), 107 sequences to the carbohydrate esterase (CE) families, 4 sequences to the polysaccharide lyases (PL) families, 268 to the glycosyl transferase (GT) families and 141 to the carbohydrate-binding module (CBM) families. Out of these 921 sequences identified as CAZymes, 142 enzymes could be potentially secreted (SignalP positive), mainly those of the GH families.

In our experiment, several CAZymes were found in the two conditions, *P. chlamydospora* and *P. oligandrum + P. chlamydospora* (Table 1). Overall, the highest number of expressed genes occurred in the pathogen + BCA treatment, with 279 vs. 230 genes for the pathogen treatment. Out of the detected CAZymes, 27 and 29 enzymes could be potentially secreted, respectively, in the *P. chlamydospora* and *P. chlamydospora* + *P. oligandrum* treatment. Interestingly, the number of expressed genes was different according to the condition. In *P. chlamydospora* + *P. oligandrum* samples, the number of GH expressed genes was higher than in the *P. chlamydospora* treatment (70 vs. 60, respectively). Inversely, for the same family, the number of expressed genes related to CAZymes was higher in the *P. chlamydospora* than in *P. chlamydospora* + *P. oligandrum* treatment. Regarding the GT family, the number of expressed genes was higher in the *P. chlamydospora* + *P. oligandrum* than in *P. chlamydospora* treatment. The CAZyme gene expression was clearly modified in the presence of the BCA at the root level.

**Figure 8:**
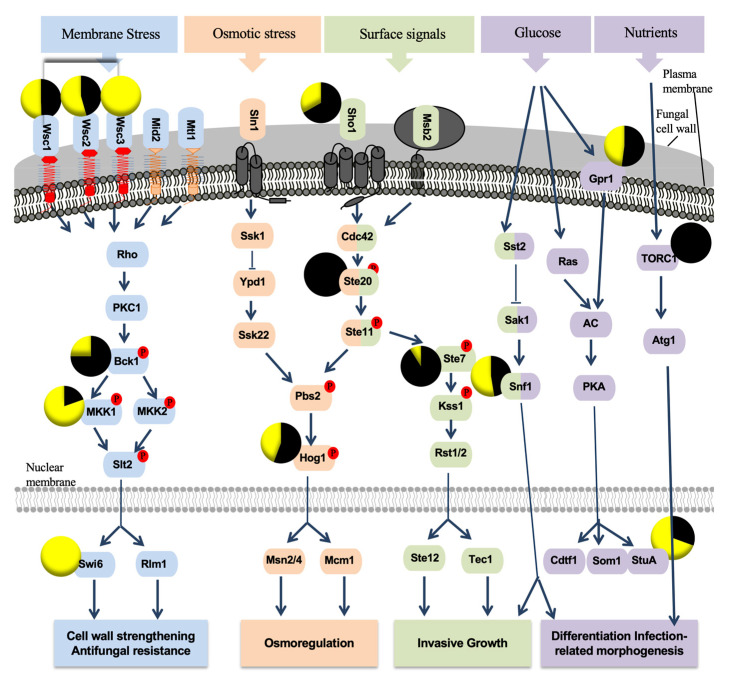
Protein kinase (PK) pathways in plant pathogenic fungi [22]. PKs are shown in colors. PKs involved in the same pathway have the same color. PKs involved in two different pathways are presented in two colors. Red circles containing the letter P indicate phosphorylated amino acid residues. Pie charts correspond to the expression level of *Phaeomoniella chlamydospora* genes encoding PKs in *P. chlamydospora* treatment (Black), and *Pythium oligandrum* + *P. chlamydospora* (yellow) treatments. Only PK genes detected in our data are shown. Expression level values are given as FPKM (fragments per kilobase per million mapped fragments). Abbreviations: AC, adenylate cyclase; PKAc, protein kinase A catalytic subunit; TORC1, target of rapamycin complex 1.

The genes potentially involved in the regulation of metabolic processes in *P. chlamydospora* were sought within BlastP annotations (against NCBI database). Their potential identifications were further tentatively confirmed by BlastP analysis against the Uniprot fungi database. In all, 34 genes were expressed in our RNAseq dataset. Out of these, 19 were present in both treatments, and 22 were expressed at a higher rate in the pathogen/BCA treatment. Most of these genes were involved in protein kinase (PK) pathways, such as cell wall remodeling, antifungal-resistance and invasive-growth (Figure 8). Most of genes involved in the “cell wall strengthening” and “Antifungal resistance” functional categories showed a higher expression in *P. chlamydospora* + *P. oligandrum* than in *P. chlamydospora* treatment (*Wsc2*, *Wsc3*, *Mkk1* and *Swi6*). However, all genes (6) involved in the “invasive growth”, “Osmoregulation” and “Differentiation Infection-related morphogenesis” functional categories were expressed at a higher level in the *P. chlamydospora* treatment (Figure 8).

**Figure 9:**
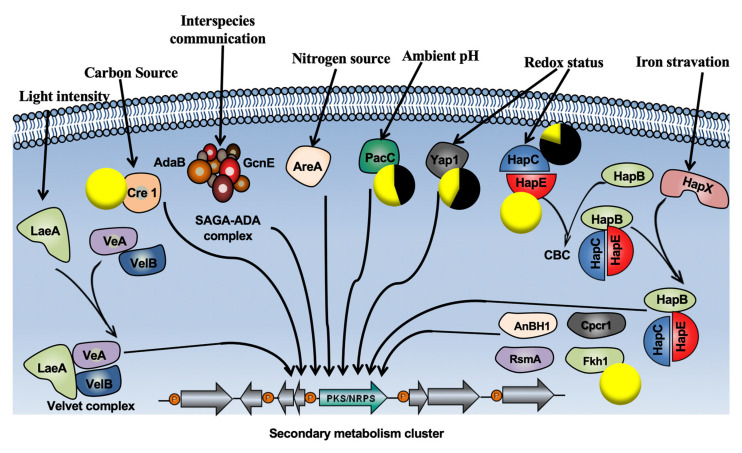
Regulatory proteins (RP) involved in the regulation of secondary metabolism gene clusters in fungi [23]. Regulatory proteins are shown in colors. Arrows and bars refer to direct interactions. Pie charts correspond to the expression level of *Phaeomoniella chlamydospora* genes encoding these RP in *P. chlamydospora* treatment (black), and *Pythium oligandrum* + *P. chlamydospora* (yellow) treatments. Only regulatory RP genes detected in our data are shown. Expression level values are given as FPKM (fragments per kilobase per million mapped fragments). Abbreviations: CBC: CCAAT-binding complex; CpcR1: cephalosporin C regulator 1; LaeA: loss of *aflR* expression A; RsmA: restorer of secondary metabolism A; SAGA-ADA: Spt-Ada-Gcn55-acetyltransferase-ADA. Reprinted by permission from Nature Publishers [24].

Six other genes were involved in a secondary metabolism regulation, such as those involved in the redox status, pH regulation and the carbon source (Figure 9). Four of those genes were implicated in redox status regulation. Interestingly, two of them (*HapE* and *Fkh1/2*) showed expression levels at 0 FPKM, when only the pathogen was present. However, in the presence of the BCA at the root level, the expression levels of these genes were strongly upregulated (Figure 9). The expression levels of the two other genes involved in the redox status (*Yap1* and *HapC*) were higher in the pathogen treatment than in the BCA + pathogen treatment. The *PacC* and *CreC* genes, involved in pH regulation and the carbon source were, respectively, more expressed in *P. chlamydospora*/*P. oligandrum* than in the *P. chlamydospora* treatment.

## 3. Discussion

*P. oligandrum* establishes a somewhat relatively complex relationship with the roots of many plants [12,13] as shown in a recent report describing the transcriptomic changes in the roots of *V. vinifera* when colonized by the oomycete [24]. The expression of several transcripts suggests that the plant sets up defense systems against the oomycete, while certain similarities with symbiotic microorganisms have also been observed [24].

In addition to its complex relationship with roots, *P. oligandrum’s* role in protecting grapevine from the attacks of many pathogens has been frequently reported [12,13]. These include a pathogenic fungus, *P. chlamydospora*, involved in esca [16,17]. It was shown that necrosis of grapevine cuttings caused by *P. chlamydospora* was significantly reduced when *P. oligandrum* colonized the plant root systems [17]. Thus, we decided to further analyze this BCA–pathogen–host interactome to throw light on how the presence of the BCA, at the root level, had led to the grapevine-trunk pathogen being less invasive. The present study showed that, by using high-throughput of transcriptome analyses, following *P. oligandrum* grapevine-root colonization, transcriptional changes occurred in the wood tissues of a perennial species such as the grapevine. Equally, transcriptional changes in a pathogenic fungus colonizing the trunk wood were, to the best of our knowledge, shown for the first time in this kind of biocontrol experiment.

### 3.1. P. oligandrum Induced Genes Involved in the Jasmonic/Ethylene Signaling Pathways

Grapevine global molecular responses were studied between 0- and 14-dpi at the trunk level. The wood samples of *P. oligandrum*-treated plants showed 189 DE genes comparing to control plants. The same result has already been observed, at the root level, in an earlier study [24]. This suggests that this type of plant response occurs throughout the whole plant, including the upper part of the grapevine.

In order to understand the specific effect of *P. oligandrum* on *P. chlamydospora*-infected grapevine responses, the expression levels of ISR-related genes in (i) mock inoculated plants, (ii) plants infected with the pathogen and (iii) plants pretreated with the BCA and infected with the pathogen, were compared. In order to understand the specific effect of *P. oligandrum* on *P. chlamydospora*-infected grapevine responses, the expression levels of ISR-related genes in (i) mock inoculated plants, (ii) plants infected with the pathogen and (iii) plants pretreated with the BCA and infected with the pathogen were compared. In all treatments, expression levels of ISR-related genes, i.e., phenylpropanoid biosynthesis, signaling hormone pathways and transcription factors, were more expressed at 0-dpi than those observed at 14-dpi. These results are in accordance with previous studies showing that grapevine defense genes are, generally, induced a few hours following biotic stress applications [25,26,27,28,29] as it is the case in our experiment where 0-dpi corresponded to 2 h after plant inoculation with the pathogen, *P. chlamydospora.*

A more detailed analysis of expression profiles revealed that certain genes involved in JA/ET pathways were more expressed in the grapevine treated with both the BCA, *P. oligandrum*, and the pathogen, than in those only infected with *P. chlamydospora* alone. Hence, it can be assumed that *P. oligandrum* promotes priming, in which the plant mobilizes its defense reactions more intensely when pathogen infection occurred. Previous studies have also demonstrated the implication of JA/ET signaling pathways in *P. oligandrum* tomato-induced resistance against other pathogens [30,31,32,33]. Genes involved in ET and JA pathways, and specifically activated in *P. oligandrum-*treated grapevine in response to *P. chlamydospora* attacks, encoded for ACC synthase, allene oxide cyclase, bHLH and AP2-ERBP transcription factors, which are involved in the regulation of disease resistance as reviewed by [34].

Moreover, Pré et al. (2008) [35] have demonstrated that the overexpression of an ORA59 gene from the AP2/ERBF family induced resistance against *B. cinerea*, and that the ORA59 mutant plants were more susceptible than control plants to infection. All these grapevine genes represent a valuable tool in characterizing the tri-partite interaction between *P. oligandrum*, *V. vinifera* and *P. chlamydospora.*

### 3.2. P. oligandrum Indirectly Modulates the Expression Level of P. chlamydospora Pathogencity Related-Genes

Characterization of *P. chlamydospora* pathogenicity related-genes, especially those involved in the colonization of the plant and effectors, is of importance. Our results showed that 463 *P. chlamydospora* recovered transcripts were identified in grapevines treated with the pathogen, whereas a higher number of transcripts, 754, were exclusively detected in grapevines treated with the BCA, *P. oligandrum*, and the pathogen, *P. chlamydospora*. Thus, the presence of *P. oligandrum*, at the grapevine root level, indirectly affected the transcriptomic responses of *P. chlamydospora*, when inoculated at the trunk level. To the best of our knowledge, this study provides the first dataset on wood fungal pathogen transcriptional changes induced by a root BCA, in which there is no contact between the two microorganisms.

Numerous studies have shown that *P. chlamydopora* produces several secondary metabolites with phytotoxic activity (i.e., toxins) [36,37,38,39]. When *P. oligandrum* colonized grapevine roots, three *P. chlamydospora* sequences encoding NRPS were not expressed, these peptides being reported as potentially responsible for the synthesis of toxic polypeptides [40]. Additionally, most t1pks genes were also more overexpressed when *P. oligandrum* was present at the root level, these genes participating in the production of the naphtelenone pentaketide toxins were found in the *P. chlamydospora* liquid culture [36,40,41]. These results evidenced the indirect effect induced by the *P. oligandrum* strain on secondary metabolite gene expression by the pathogen. It can be hypothesized that the potential regulation of specific transcripts encoding for these toxins corresponds to a stress state of the pathogen induced by the presence of the BCA, *P. oligandrum*, at the root level.

CAZymes transcripts are also affected. CAZymes are proteins that catalyze the breakdown, biosynthesis or modification of carbohydrates and glycoconjugates [42,43,44,45]. Differences in the expression levels of *P. chlamydospora* CAZyme-related transcripts detected in our dataset were observed, between treatments. Results obtained in our study also revealed that the secretion of the expression level of *P. chlamydospora* genes encoding for GHs, involved in the degradation of cellulose and hemicelluloses, was greatly reduced in the presence of *P. oligandrum* at the root level. On the contrary, the GTs responsible for the biosynthesis glycosidic bonds from phospho-activated sugar donors [45] were more secreted. Accordingly, it could be supposed that, as the trunk pathogen is stressed in grapevines treated with *P. oligandrum*, it is unable to progress *in planta* and it needs to secrete enzymes to obtain a source of energy. Overall, these results confirmed the indirect effect of *P. oligandrum* root inoculation on the *P. chlamydospora* transcriptome at the trunk level.

The *P. chlamydospora* transcripts of genes related to several proteins important for the pathogen–host relationship, namely transcription factors and protein kinases [22,23], were identified. Several of the above-mentioned protein genes, especially those involved in the cell wall strengthening pathway (*Wsc* family, *Swi6* gene) and regulation of the redox status (*HapE* and *Fkh1* genes), presented a high level of expression in *P. chlamydospora* + *P. oligandrum*, than in the *P. chlamydospora* one. One explanation could be that effectors, produced by *P. oligandrum* at the root level, were transported via the grapevine vascular system and induce stresses on *P. chlamydospora*, when they reach the trunk level.

Other *P. chlamydospora* genes possibly involved in invasive growth, *Ste7*, *Ste20* and *Sho1*, showed the highest expression levels in the pathogen condition. In the presence of *P. oligandrum*, expression levels of these three genes were strongly reduced and when combined with other factors, it may be involved in reducing the wood necroses caused by *P. chlamydospora*.

To conclude, our data showed that during the tripartite interaction grapevine + *P. chlamydosporum* and *P. oligandrum*, the JA/ET signaling pathways are promoted with certain genes being more induced when plants were pretreated with the BCA, *P. oligandrum*, thus highlighting a priming effect. The indirect effect of *P. oligandrum* on the *P. chlamydospora* transcriptome was also demonstrated, which contributes to a better understanding of the protection mechanisms induced by the potential oomycete-BCA.

## 4. Methods

### 4.1. Plant and Microorganism Materials

In order to choose the best samples to characterize the tripartite interaction between *P. oligandrum*, the grapevine and *P. chlamydospora*, using high throughput transcriptomic techniques such as a microarray and RNAseq, a preliminary study has been already carried out [17]. In that previous study, three inocula of *P. oligandrum* were assessed to protect the grapevine against *P. chlamydospora*. Plant responses were evaluated using a set of 22 genes globally involved in grapevine defenses against different biotic stresses. For the present study, depending on our previous results [17], we selected the samples showing the *P. oligandrum* mean root colonization level of 39% (*P. oligandrum*, inoculum Po2), and the highest plant protection level (50%) against the pathogen (*P. chlamydospora*, strain SO37). The different control treatments were integrated in the analysis.

The assay was conducted in a greenhouse and at 7–8 leaf stage, grapevine plants (*V. vinifera* L. cv. Cabernet Sauvignon) were infected with *P. chlamydospora* (strain SO37, INRA-UMR SAVE collection, Bordeaux, France) at the stem level. *P. oligandrum* root inoculation (at 2 × 10^4^ oospores per mL suspension) was performed twice at intervals of 4 days, with the first inoculation being carried out 7 days before pathogen infection, and the second one 3 days before. The *P. oligandrum* inocula were composed of an oospore-mycelium homogenate of a mixture of two strains (Sto-1 and Oth-4), they were prepared by Biovitis (Saint-Étienne-de-Chomeil, France) as described previously [46].

The experimental design consisted of five conditions composed of grapevines (i) inoculated on roots with *P. oligandrum*; (ii) infected in the wood trunk by the pathogenic agent *P*. *chlamydospora*; (iii) pretreated on roots with *P. oligandrum* and then infected with *P. chlamydospora*; (iv) mock inoculated (cuttings with a hole) and (v) control (cuttings without any treatment).

For the transcriptome analyses, the stems of nine grapevines per treatment were collected, at each sampling time point, and pooled on three replicates (three plants per replicate) in order to reduce biological variation-related noise. For each plant, collected wood samples were measured at about 4 cm (2 cm above and 2 cm below the inoculation point). Collected materials at 0-dpi (2 h post pathogen inoculation) and 14-dpi (14 days post pathogen inoculation) were immediately frozen in liquid nitrogen and stored at −80 °C until use.

### 4.2. RNA Extraction

Grapevine wood samples were ground in liquid nitrogen, and 200 mg aliquots were used for RNA extraction. A commercial RNA extraction kit (RNeasy Plant Mini Kit, Qiagen, Hilden, Germany) was employed, with modifications as previously described [17]. Total RNA samples were subjected to standard quality controls: Nanodrop (ND-1000, ThermoScientific, Waltham, MA, USA) for all samples and the BioAnalyzer (Agilent, Carpinteria, CA, USA) for the microarray and RNAseq samples. All samples were stored at −80 °C before use in RT-qPCR experiments, or else stored at −80 °C before being shipped in dry ice, either to the GeT (Genome & Transcriptome) platform (Toulouse, France) for subsequent microarray analyses, or to the IGBMC (Institut de génétique et de biologie moléculaire et cellulaire) sequencing platform (Strasbourg, France) for RNAseq analyses.

### 4.3. Microarray Analyses, Data Processing and Deposition

The microarrays used were the grapevine whole-genome microarrays by Nimblegen, Roche, Basel, Switzerland (Design name 090918 Vitus exp HX12). Microarray hybridizations were performed by the Get Platform (Toulouse, France) in accordance with the manufacturer’s instructions. The microarray data were analyzed using the statistical package R, version 2.14.0 with various Bioconductor packages [47] (http://www.bioconductor.org). Microarray quality controls were performed using the ArrayQualityMetrics package [48]. Expression intensities were background corrected, quantile-normalized and summarized using the RMA function of the oligo package [49]. The raw and normalized microarray data are available in the ArrayExpress database under accession number E-MTAB-12345.

Differentially expressed (DE) genes were identified using the Limma package [50] at a *p* value < 0.01 using the MultiExperiment Viewer software MeV [51]. The log_2_ of the fold change (FC) was used to obtain contrastingly regulated genes between 0 and 14 dpi, for each treatment.

### 4.4. RNAseq Analyses

RNAseq library preparation was performed using an rRNA-depletion kit (RiboZero, Qiagen, Hilden, Germany), as recommended by the manufacturer. Sequencing was performed by the IGBMC Microarray and Sequencing platform, a member of the “France Génomique” consortium (ANR-10-INBS-0009). The libraries were sequenced on an Illumina Hiseq 2500 as paired-end 100 base reads, in accordance with Illumina’s instructions. Image analysis and base calling were performed using RTA 1.17.21.3 and CASAVA 1.8.2. Files containing raw data (fastq files) obtained from the IGBMC Sequencing platform were transferred to the bioinformatics servers of the GenoToul bioinformatics facility and analyzed remotely by connecting to the linux cluster. The procedure that we followed for RNAseq analyses has already been described [52]. RNAseq data are available in the ArrayExpress database under accession number E-MTAB-3966. The widely used computational tool Antismash [53] to identify different gene clusters involved in pathogen secondary metabolite biosynthesis.

### 4.5. Reverse Transcriptase Quantitative PCR Experiments

RNA retro-transcription was performed with the Superscript III kit from Invitrogen (using oligo(dT) primers, 1.5 µg of RNA and in accordance with to the manufacturer’s instructions). Gene expression was analyzed on a Stratagene MX3005P PCR machine (Agilent Technologies), with a kit MESA BLUE qPCR for SYBR Assay (Eurogentec, Liége, Belgium). Each reaction was performed in duplicate, using 1 µL of each primer (forward and reverse) at 1 µM, 7 µL of the fluorescein mix and 5 µL of cDNAs [54]. All qRT-PCR reactions were performed as follows: 95 °C for 10 min, followed by 40 cycles of 95 °C for 3 s and 60 °C for 30 s. Determination of the relative quantity of the target gene transcript was performed using the 2^−ΔΔCT^ method [55].

The raw data were exported using the software from the PCR equipment, and then analyzed using the LinRegPCR software [56]. Primers were designed on the NCBI (National Center for Biotechnology Information) website using the Primer-Blast utility [57]. The sequences used for RT-qPCR and corresponding primers are given in Table S1, and those used as references are given in Table S2. The comparison between the microarray and qPCR levels of expression was carried out using regression analysis (Figure S1).

## Figures and Tables

**Table 1:** CAZymes genes predicted in the *Phaeomoniella chlamydospora* genome and transcripts detected in the RNAseq samples. Within parentheses: the number of genes related to CAZymes that could be potentially secreted (SignalP positive). Pch: *P. chlamydospora*; PoPch: *P. oligandrum* and *P. chlamydospora*.

Families	Number of Genes Predicted in the Genome	Number of Genes Detected in Samples	
		Pch	PoPch
Glycoside hydrolases (GH)	345 (72)	60 (17)	70 (9)
Glycosyl transferases (GT)	268 (17)	34 (3)	69 (7)
Carbohydrate-binding module (CBM)	141 (26)	27 (6)	37 (4)
Carbohydrate esterases (CE)	107 (12)	13 (1)	10 (3)
Auxiliary Activities (AA)	56 (14)	9 (0)	7 (2)
Polysaccharide Lyases (PL)	4 (1)	2 (0)	1 (1)

## Supplementary Materials

Supplementary materials can be found at https://www.mdpi.com/1422-0067/21/18/6876/s1. Figure S1. Comparison of microarray and qPCR expression levels Log2 (FC) by regression analysis. Table S1. Genes used for microarray validation by RT-qPCR. Table S2. Genes used as reference in RT-qPCR analyses.
