# Supplementary material for: The Biocontrol Root-Oomycete, Pythium Oligandrum, Triggers Grapevine Resistance and Shifts in the Transcriptome of the Trunk Pathogenic Fungus, Phaeomoniella Chlamydospora
Source: Int J Mol Sci. 2020 Sep 19;21(18):6876. doi: 10.3390/ijms21186876 (PMC7555917; doi:10.3390/ijms21186876)
Supplement: Supplementary file 1 [file ijms-21-06876-s001.pdf]

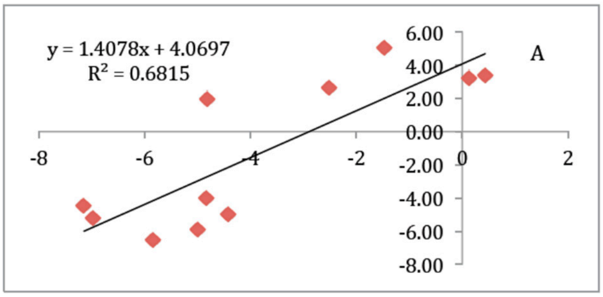

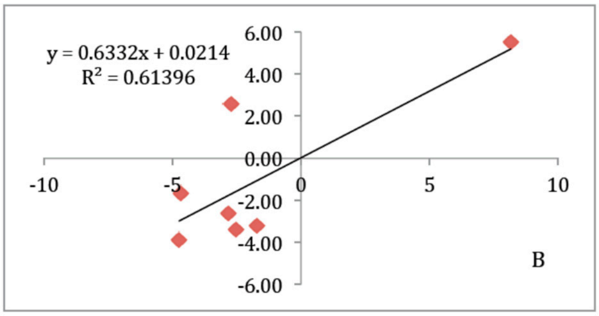

Supplementary Figure 1 Comparison of microarray and qPCR expression levels Log_2 (FC) by regression analysis. (**A**) Samples treated with *Pythium oligandrum* Po2 and *Phaeomoniella chlamydospora* SO37; (**B**) Mock-inoculated plants. Each red point represents one studied gene (related to ABC transporter, auxin binding protein, cytochrome P450, expansin, GDSL lipase, dioxygenase (gibberelin), glycosyl transferase, auxin responsive protein, naringenin dioxygenase, polygalacturonase, terpene synthase et glycosyl transferase for the A condition/ABC transporter, allene oxyde cyclase, cytochrome P450, expansin, dioxygenase (gibberelin), auxin responsive protein, polygalacturonase, phospholipase et glycosyl transferase for the B condition.

Supplementary Table 1 Genes used for microarray validation by RT-qPCR.

Gene identifier* V0 - 12X V1 - 12X	Name	Mapman functional category	Sens (5'>3')	Anti-sens (3'>5')
chr11_jgvv16_101_t01 Vv11s0016g01220	Auxin binding protein	20.2.99stress.abiotic.unspecif ied	CAAGTGAATGGGGCTGGGA T	AAGGATCTGGAGACCAGG CT
chr18_jgvv1_77_t01 Vv18s0001g01130	Expansin	10.7cell wall.modification	GGTGGACTAAAGCTCACGCT	AATGCTGTGCTCAAAGCTG C
chr5_jgvv20_35_t01 Vv05s0020g00420	Polygalacturona-se	10.6.3cell wall.degradation.pectate lyases and polygalacturonases	TCAACAATCTTCTCCGGCCC	TTCCGCTTCCTGTGAAGGT C
chr7_jgvv31_232_t01 Vv07s0031g02550	ABC transporter	34.16 transport.ABC transportersand multidrug resistance systems	TTCGTGGCTCAGAAGGACAC	ATCAACTGTGCCGGAGGA AG
chr14_jgvv66_158_t01 Vv14s0066g01730	Glycosyl transferase	10.2 cell- wall.cellulose synthesis	GTCCTACATGGCTTGGCTGT	CGGGCTTCAACTTCTTGTG C
chr1_jgvv11_349_t01 Vv01s0011g03090	Allene oxide cyclase	17.7.1.4 hormone metabolism.jasmonate.synth esis degradation.allene oxidase cyclase	CTGAGTCAGAAGCCAGCCA A	CTCCAGGTCTCCGGTGTAG A
chr10_jgvv3_374_t01 Vv10s0003g04200	Phospholipase	11.9.2.1 lipid metabolism.lipid degradation.lipases.triacylgl ycerol lipase	TCTGGCTTCAGTTGGAGTGC	GCTGATTCCGGAGAGCTGT T
chr13_random_jgvv123_27_t 01 Vv13s0067g03700	Terpenoid Synthase	16.1.5 secondary metabolism.isoprenoids.terp enoids	ATGGAGATGGGCATGGTGTT C	TGACAACTTTCTAGACCAG GGG
chr18_jgvv1_338_t01 Vv18s0001g06090	Glycosyl transferase	26.2 misc.UDP glucosyl and glucoronyl transferases	GGCCTTGGAGTGGAAGACTC	TCGCTGAGTTGGGACATTC G
chr19_jgvv140_8_t01 Vv19s0140g00120	Dioxygenase	17.6.1.13 hormone metabolism.gibberelin.synth esis degradation.GA2 oxidase	ACGTGTTCTTCCAAAGCCCT	TGGCAGGCTTCAATGAGG AG
chr2_jgvv25_272_t01 Vv02s0025g02960	Dioxygenase	17.5.1 hormone metabolism.ethylene.synthes is degradation	CGCAACCAGAGCTGACCTAT	TTAAGACCTGCAACCCAG GC
chr3_jgvv91_89_t01 Vv03s0091g00310	Auxin responsive protein	17.2.3 hormone metabolism.auxin.induced regulated responsive activated	CCGAGAAGGATGCAAAAGC G	GACCGTTGACCCTTCCAGT T
chr6_jgvv80_106_t01 Vv06s0080g00090	Cytochrome P450	26.10 misc.cytochromeP450	CTTTCTCGGCCATCTTGGGT	GAGCTCCAGACTCGCCATT T
chr9_jgvv2_42_t01 Vv09s0002g00570	GDSL lipase	26.28 misc.GDSL motif lipase	TAGGGCCTCTGGGTTGTGTA	TTTGAATCTGGGTAGGCGG G

* *Vitis vinifera* sequencing has produced several versions: 8X, 12X V0, 12X V1

Supplementary Table 2 Genes used as reference in RT-qPCR analyses

Gene identifier * V0 - 12X V1 - 12X	Name	Mapman functional category	Sens (5'>3')	Anti-sens (3'>5')
chr17_jgvv0_5_t01 Vv17s0000g10430	Glyceraldehyde-3-phosphate dehydrogenase	4.9 glycolysis.glyceraldehyde3-phosphate dehydrogenase	GGCGTCGCCCAGATCGTGAT	AGGCCGAGAAGGGTGACCGG
chr5_jgvv20_398_t01 Vv05s0020g04250	Mitosis protein dim1	35.1 not assigned.no ontology	ACCTTCACTCTGGATGGGCCGT	CCCAATCGTGGCCGAACCGA
chr6_jgvv4_524_t01 Vv06s0004g02820	Sand protein	35.1 not assigned.no ontology	CGGGGTCGCAAACGGATCGT	GCCCCGTTCCCCAGCATAGC

* *Vitis vinifera* sequencing has produced several versions: 8X, 12X V0, 12X V1
